# A Rare Case of Bilateral Patellar Tendon Ruptures: A Case Report and Literature Review 

**DOI:** 10.1155/2016/6912968

**Published:** 2016-04-20

**Authors:** Nadim Tarazi, Padhraig O'loughlin, Amin Amin, Peter Keogh

**Affiliations:** Department of Trauma & Orthopaedics, Connolly Hospital, Blanchardstown, Dublin, Ireland

## Abstract

Bilateral patellar tendon ruptures are rare. The majority of case reports describing bilateral patellar tendon ruptures have occurred in patients with predisposing factors to tendinopathy. We describe a case of bilateral patellar tendon rupture sustained following minimal trauma by a patient with no systemic disease or history of steroid use. Due to the rarity of this injury, clinical suspicion is low. It is reported that 38% of patellar tendon ruptures are misdiagnosed initially. Therefore careful history taking and physical examination is integral in ensuring a diagnosis is achieved for early primary repair. We discuss the aetiology of spontaneous tendon rupture and report a literature review of bilateral patellar tendon ruptures.

## 1. Introduction

Bilateral patellar tendon ruptures are rare, especially amongst patients with no predisposing factors for tendinopathy [1]. The majority of case reports present in the literature occurred in patients with predisposing factors to tendinopathy [2]. We describe a case of bilateral patellar tendon rupture sustained following minimal trauma by a patient with no systemic disease or history of steroid use.

## 2. Case History

We present the case of a 45-year-old healthy man. He tripped on a step while unloading heavy boxes at work and fell forward with his right knee in flexion leading to severe pain. There was no definite direct blow to the knee. He attempted to stand up using his left leg but it also gave way. He subsequently presented to the emergency department unable to mobilize due to severe pain in both of his knees.

The patient had no significant relevant past medical history such as rheumatoid arthritis, Diabetes Mellitus, or connective tissue disease that might predispose him to tendon injury. He is a nonsmoker and has never been administered long-term steroids. On examination, he had two visibly swollen knee joints without any gross skin defects or ecchymosis. He was maximally tender at the anterior aspects of both knees with readily palpable infrapatellar tendinous defects. He was unable to straight leg raise on either side.

**Figure 1:**
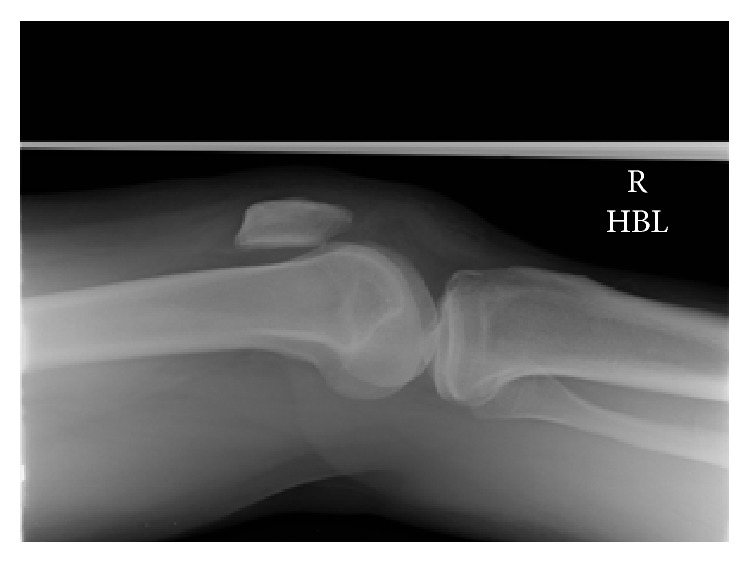
Lateral radiographs of right knee.

**Figure 2:**
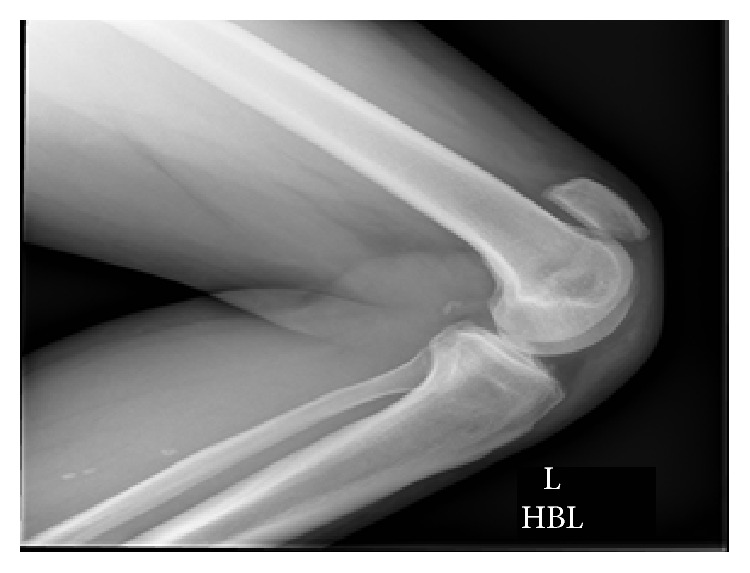
Lateral radiograph of left knee.

Plain radiographs showed bilateral superior patellar displacement (Figures 1 and 2).

The diagnosis of bilateral patellar tendon rupture was made based on the clinical and radiological findings above. Surgery was performed on day one after injury. Intraoperatively, both patellar tendon bodies were uninjured but avulsed off the lower pole of each patella. There was evidence of bilateral retinacular tears with no signs of synovitis. Biopsies of the synovium and tendon were sent for histology. Three drill holes were made from the inferior pole to the superior pole of each patella with a 2.0 mm drill bit. Number two and five Ethibond (Ethibond Inc., Somerville, NJ, USA) sutures were used to perform a Krackow repair. A suture passer (superior to inferior) was used to pass the suture ends. The two pairs of knots over patella were tied. The retinacular tears were then repaired using 2,0 Vicryl. The repair was augmented using a tension band wire.

**Figure 3:**
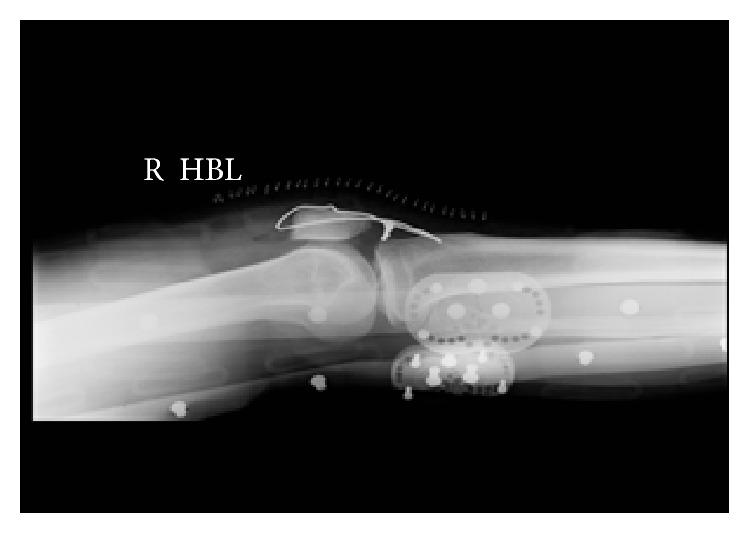
Postoperative right knee radiograph.

**Figure 4:**
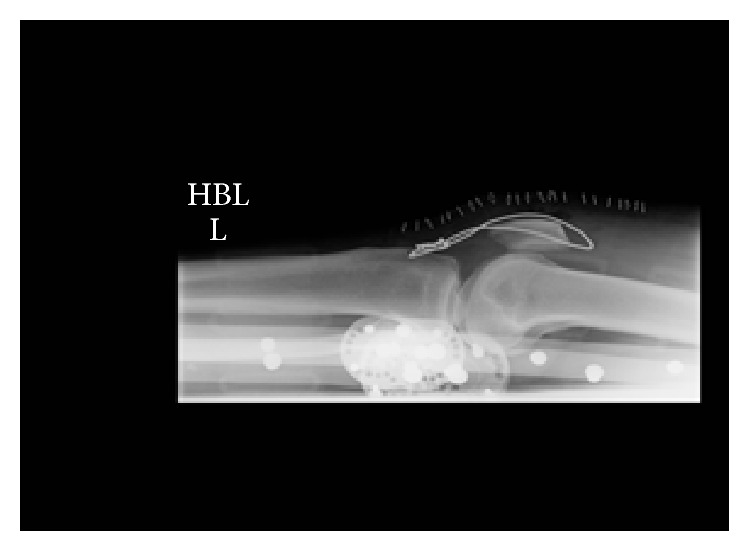
Postoperative left knee radiograph.

Postoperatively he was placed in Don-Joy (DJO Global, Vista, CA, USA) braces locked in extension and was allowed to mobilize with physiotherapy, weight-bearing as tolerated with the support of crutches. His post-op radiographs are shown in Figures 3 and 4.

The histology of the specimens sent was consistent with acute bilateral patellar tendonitis.

This patient had an uncomplicated perioperative course. He was placed in a Don-Joy (DJO Global, Surrey, UK) brace locked in extension for two weeks and he was allowed to bear weight as tolerated. At two-week follow-up, the brace was adjusted to permit movement between zero and 30 degrees with a gradual increase by 30 degrees every two weeks. The Don-Joy brace was removed at 12 weeks postoperatively. In relation to his physiotherapy, his ROM was gradually increased every 2 weeks and was weight-bearing as tolerated while in 20-degree flexion. Between six and 12 weeks, there was a gradual progression to weight-bearing with knee flexion with initial avoidance of weight-bearing with knee flexion greater than 70 degrees, until a full 12-week postsurgery had elapsed. At one year post-op, the patient had a full range of motion of his knee joint and underwent removal of the tension band wire under general anaesthetic. His final follow-up was at his first post-op visit having had all metal-work removed at 12 months post-op. At that visit, it was noted that both wound sites had united satisfactorily and had a full range of movement of his knees bilaterally. At this point, it was decided that the patient did not need to return for regular follow-up.

## 3. Discussion

The patellar tendon is one of the most important tendons in the human body as it forms an integral part of the extensor mechanism of the knee which has an essential role in gait. The extensor mechanism consists of the quadriceps muscles and their tendon, the patella, and patellar tendon which attaches to the tibial tuberosity. Rupture of the patellar or quadriceps tendon or a fracture of the patella itself can disrupt the extensor mechanism [3].

Patellar tendon ruptures are typically unilateral and tend to occur in athletic adults under the age of 40. In the presence of a healthy patellar tendon, the patella is considered to be the weakest link in the extensor mechanism and tensile overload usually leads to a transverse fracture of the patella [4]. The usual mechanism of injury is knee flexion combined with quadriceps contraction. A healthy normal patellar tendon requires a massive force to be disrupted and should not rupture under physiological loads [5]. It is estimated that 17.5 times the body weight is needed to cause rupture of a normal patellar tendon [6]. It is well documented in the literature that patients with conditions such as systemic lupus erythematosus (SLE), Diabetes Mellitus, rheumatoid arthritis, chronic renal failure, and corticosteroid use are susceptible to patellar tendon ruptures as these conditions are known to weaken collagenous structures [7, 8]. Jozsa and Kannus defined a spontaneous rupture of a tendon as a rupture that occurs during movement and activity that should not and usually does not damage the involved musculotendinous units [9]. The aetiology of spontaneous bilateral patellar tendon rupture is still not clearly understood, but it is believed that patellar tendon ruptures that occur in indirect trauma are considered the end stage of long standing chronic tendon degeneration, secondary to repetitive microtrauma.

Kanus and Józsa evaluated specimens from the biopsy of ruptured tendons in 891 patients. These specimens were compared with 445 tendons taken at the time of death from the cadavera of previously healthy individuals who died accidentally. Both groups of tendons underwent histopathological analysis using polarised light and electron microscopy. There were characteristic histopathological patterns in the spontaneously ruptured tendons. 97% of the pathological changes were degenerative and included hypoxic degenerative tendinopathy, mucoid degeneration, tendolipomatosis, and calcifying tendinopathy, either alone or in combination, although these changes were also found in 34% of the healthy unruptured tendons. Therefore, the finding indicated that degenerative changes are common in the tendons of people who are older than thirty-five years and that these changes are associated with spontaneous ruptures [10].

Järvinen et al. analyzed collagen fibre thickness and crimp formation in healthy and ruptured tendons. They examined 66 spontaneously ruptured tendons and were compared to healthy nonruptured tendon. The results showed that spontaneously ruptured tendons display regions with decreased collagen fibre thickness, decreased crimp angle, and disrupted crimp continuity, microscopic alterations that possibly result in reduced strength of tendons being less resistant to tensile forces and thus place them at increased risk of ruptures [11].

We found our case of particular interest for multiple reasons. Firstly, our patient had no predisposing risk factors for bilateral tendon rupture. Secondly, the trauma he sustained was not of significant force and was unilateral. Finally, his histology showed acute inflammatory changes to both tendons with no evidence of chronic tendonitis.

Due to the rarity of bilateral patellar tendon ruptures, clinical suspicion is low. Siwek and Rao reported that 38% of patellar tendon ruptures were misdiagnosed initially. Diagnosis could be even more challenging in cases with severe hematoma that very often accompanies acute rupture and may conceal the important diagnostic signs, such as a palpable infrapatellar gap. For these reasons the correct diagnosis may be missed and it is of utmost importance that early diagnosis of patellar tendon ruptures is established [12].

## 4. Conclusion

In conclusion, this young, healthy male patient sustained bilateral patellar tendon ruptures due to large but indirect traumatic forces. The authors are happy to report that he ultimately made an excellent recovery following open repair with additional tension-band-wiring, a step-wise, focused rehabilitation protocol and removal of hardware at 12 months after surgery. He has now been discharged from regular follow-up.
